# Ehlers-Danlos Syndrome: A Tale of Two Cases Highlighting Rare Subtypes and Diagnostic Considerations

**DOI:** 10.7759/cureus.93109

**Published:** 2025-09-24

**Authors:** Karubaki Pati, Saumya R Tripathy, Sarit S Patnaik, Manoj K Parida, Bidyut K Das

**Affiliations:** 1 Rheumatology, Institute of Post Graduate Medical Education & Research, Kolkata, IND; 2 Rheumatology, Srirama Chandra Bhanja Medical College and Hospital (SCBMCH), Cuttack, IND

**Keywords:** adducted thumb-clubfoot syndrome, ehlers-danlos syndrome, hypermobile eds, hypermobility syndrome, musculocontractural ehlers–danlos syndome

## Abstract

Ehlers-Danlos syndrome (EDS) comprises a group of rare connective tissue disorders marked by multisystem involvement and considerable clinical variability. This report presents two cases of young individuals with complex medical histories involving joint hypermobility, musculoskeletal deformities, ocular abnormalities, and unexplained cardiovascular symptoms. Both cases were initially misclassified under more common subtypes of EDS. However, comprehensive clinical evaluation and application of updated classification criteria led to a re-evaluation of the initial diagnoses. These cases highlight the diagnostic challenges posed by EDS and emphasize the need for heightened clinical suspicion and systematic assessment when encountering atypical presentations.

## Introduction

Ehlers-Danlos syndrome (EDS) is a heritable connective tissue disorder that is clinically and genetically heterogeneous and has multiple subtypes [1]. Eleven subtypes were described for the first time by the 1988 Berlin Nosology [2]. Subsequently, with the discovery of the molecular and biochemical basis of EDS, the nosology was revised in 1998, and six subtypes were described as “Villefranche Nosology” [3]. With the advent of next-generation sequencing, genetic mutations responsible for most EDS subtypes have been discovered. Additionally, various novel clinical subtypes with varying genetic and molecular mechanisms have been described. Based on this, 13 subtypes have been described under the latest classification system, “the 2017 International Classification for the Ehlers-Danlos Syndromes” [4]. Each subtype has major and minor criteria and has defined genetic markers except for hypermobile EDS [4]. EDS and all its subtypes are characterized by defects in the connective tissue, resulting in hypermobile joints, hyper-flexible skin, and fragile tissues. Patients with hypermobile joints suffer from chronic aches and pains, recurrent sprains, and subluxation or dislocation of joints, which affects their personal and professional lives. 

In addition to the above clinical features, classical EDS may present with easy bruising, soft, doughy skin, skin fragility, molluscoid pseudo-tumors, subcutaneous spheroids, hernia, and epicanthal folds. More than 90% of classical EDS are characterized by mutation in type V collagen (COL5A1 and COL5A2) [4]. Classical EDS is autosomal dominant and often has a history of an affected first-degree relative. In contrast, the musculoskeletal variant is an autosomal recessive mutation documented in the HST14 or dermatan sulfate epimerase (DSE) genes. It is characterized by congenital multifocal contractures and craniofacial abnormalities evident at birth or in infancy. At the other spectrum of EDS variants is the hypermobile EDS variant, in which the genetic defect is yet to be determined. This is the mildest clinical variant and is autosomal dominant in inheritance.

Herein, we describe two rare phenotypes of Ehlers-Danlos syndrome that were diagnosed based on clinical features.

## Case presentation

Case report one

**Figure 1:**
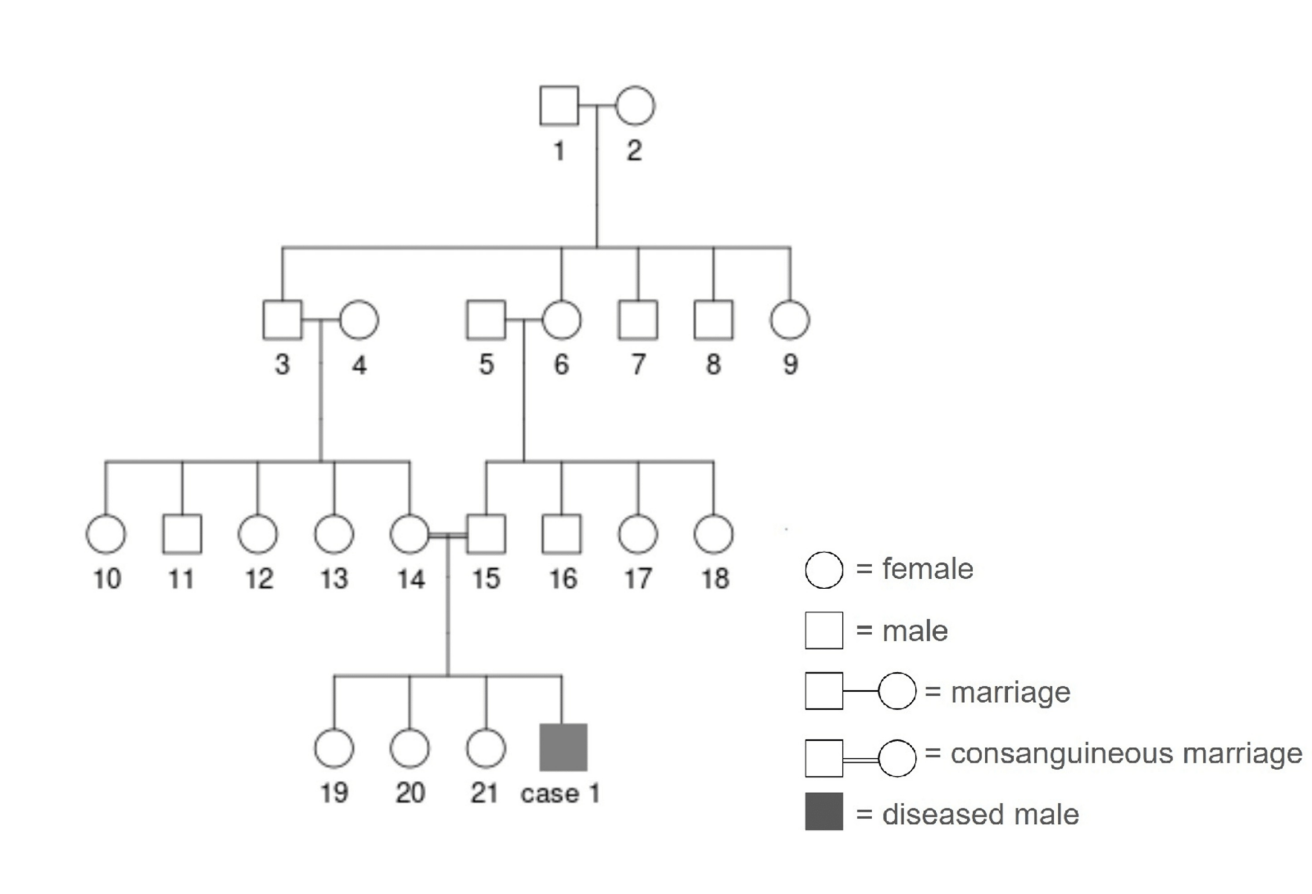
Case one family tree showing consanguinity between 1st cousins 3 – grandfather of case 1, 6- grandmother of case 1, 14- mother of case 1, 15- father of case 1; 3 and 6 were siblings, thus making the parents of case 1 first cousins.

A 16-year-old boy presented to the rheumatology outpatient department (OPD) with the chief complaint of a large swelling around the left knee following a trivial trauma that occurred one month before presentation at the hospital. The patient was born to a consanguineous marriage. The family tree of the patient is shown in Figure 1.

**Figure 2:**
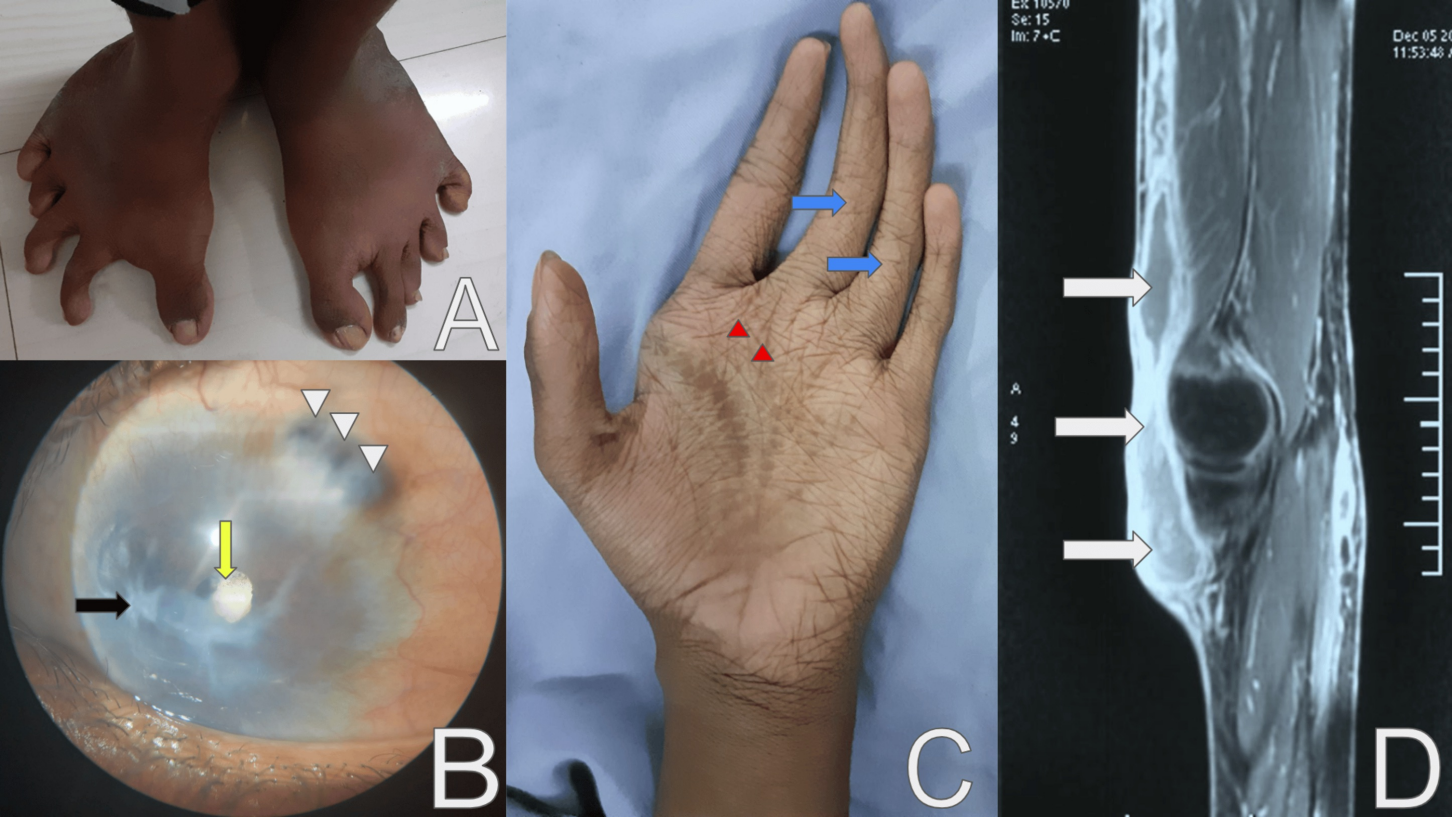
Case one findings A: bilateral congenital clubfeet; B: ciliary staphyloma in upper temporal quadrant (white triangles), corneal opacification (black solid arrow) and cataract (yellow arrow) in the left eye; C: spider-like long and slender digits (arachnodactyly; blue solid arrows) and increased palmar creases (red triangles); D: MRI of left knee showing hematoma extending from the anterior of mid-thigh to upper third of the leg (white solid arrows).

**Figure 3:**
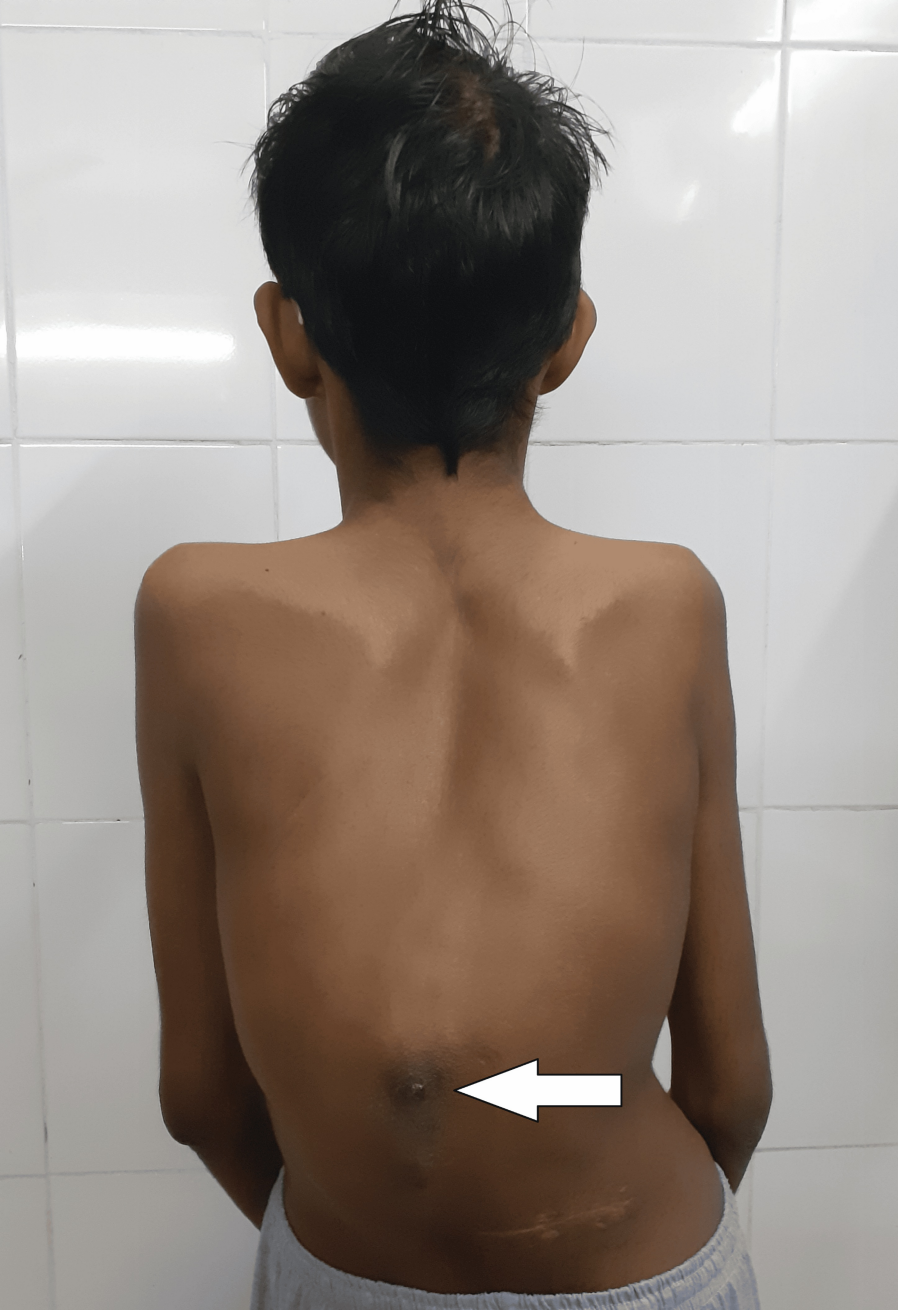
Case 1: kyphoscoliosis with concavity to right (white arrow)

He had a history of bilateral congenital clubfoot for which he underwent corrective surgery at the age of six years (Figure 2A). Following ocular trauma at the age of seven years, he developed corneal opacification in the left eye (Figure 2B). The right eye had high myopia and glaucoma, for which he underwent trabeculectomy at the age of 14 years. There was no similar family history. On examination, he had hyperextensible skin, arachnodactyly, increased palmar creases, and papyraceous scarring of the skin (Figure 2C). There was swelling over the left knee, extending from the mid-thigh to the mid-calf. The skin over the swelling had normal temperature, was non-tender, and indurated. The joints were hypermobile, as evidenced by a Beighton’s score of 6 and kyphoscoliosis with concavity to the right. The visual acuity of the left eye was limited to the perception of light. In addition, the left eye also had a ciliary staphyloma suggestive of a weak globe, stromal corneal opacification, and traumatic cataract. The right eye had an uncorrected visual acuity of 6/36, with the fundus suggestive of high myopia and advanced glaucoma. The anteroposterior (AP) axial length of the eyeball was increased (30 mm) on MRI. The hematoma, for which the patient initially presented, was found to extend from the mid-thigh to the upper third of the leg on MRI (Figure 2D). The patient also had a history of bilateral cryptorchidism and kyphoscoliosis (Figure 3).

**Figure 4:**
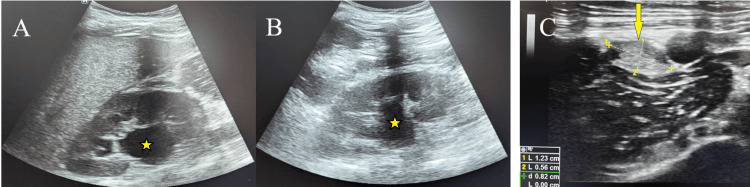
Case 1: abdominal and pelvic ultrasound Abdominal ultrasound showing dilated pelvicalyceal system (yellow star); A: right kidney; B: left kidney; C: ectopic testis in the right inguinal canal (yellow arrow).

In addition, there was evidence of bilateral avulsion of the tibial tuberosity (Osgood-Schlatter disease) on the radiographs. Abdominal and pelvic ultrasonography revealed bilateral hydroureteronephrosis (Figures 4A, 4B) with cryptorchidism (Figure 4C). Routine investigations like complete blood counts, prothrombin time, activated partial thromboplastin time, international normalised ratio, liver and kidney function tests were within normal limits, and inflammatory markers were tested, which showed normal ESR and a mildly elevated C-reactive protein (CRP) (Table 1).

**Table 1:** Routine blood investigations of case one Results were within normal limits, except mildly elevated CRP. ESR: erythrocyte sedimentation rate; CRP: C-reactive protein; ALP: alkaline phosphatase; SGOT: serum glutamic-oxaloacetic transaminase; SGPT: serum glutamic-pyruvic transaminase; PT: prothrombin time; INR: international normalized ratio; aPTT: activated partial thromboplastin time.

Parameters	Value (normal range)
Hemoglobin	13.4g/dL (13-17g/dL)
White blood cell	9300/mm3 (4000-11000/mm3)
Differential count	Neutrophils 58% Lymphocytes 36% Eosinophils 4% Monocyte 2% Basophils 0%
Platelet count	2.5lacs/mm3 (1.5-4.5/mm3)
ESR (Westergren)	16 (0-15mm 1st hour)
CRP	4 (0.3-3mg/L)
Blood urea	19 (7-20mg/dL)
Serum creatinine	0.8 (0.6-1.4mg/dL)
Serum sodium	136 (135-145meq/L)
Serum potassium	3.6 (3.5-5.1meq/L)
Total bilirubin	0.2 (0.1-1.2mg/dL)
Serum ALP	102 (44-147U/L)
SGOT	16 (10-40U/L)
SGPT	18 (10-40U/L)
Serum total protein	6.2 (6-8g/dL)
Serum albumin	3.6 (3.5-5g/dL)
PT	13 (10-14 seconds)
INR	1.13
aPTT	27 (25-35 seconds)

Owing to recurrent bleeding manifestations and associated cryptorchidism, he was initially diagnosed with vascular Ehlers-Danlos syndrome. However, the patient had never experienced any catastrophic event due to vascular rupture to date.

However, on application of the 2017 International Classification for the Ehlers-Danlos Syndromes, we realized that the case was a musculocontractural variant of EDS (mcEDS). Musculocontractural is one of the rarer varieties, with an autosomal recessive pattern of inheritance [4]. The criteria for classifying mcEDS are listed in Table 2, which includes three major and 15 minor criteria [4].

**Table 2:** Case 1 and the criteria for musculocontractural EDS Minimal criteria suggestive of mcEDS: a) At birth or in early childhood - major criteria (1) congenital multiple contractures AND (2) characteristic craniofacial features; b) In adolescence and in adulthood - major criteria (1) congenital multiple contractures AND (3) characteristic cutaneous features. # Criteria adapted from the 2017 International Classification of the Ehlers–Danlos Syndromes [4]. AR: autosomal recessive; EDS: Ehlers-Danlos syndrome.

musculocontractural EDS#	Case 1
Inheritance: Autosomal recessive	Being born to consanguineous parents without a history of a similar disorder in any other family member is consistent with the AR pattern of inheritance
Major criteria	
1. Congenital multiple contractures, characteristically adduction-flexion contractures and/ or talipes equino-varus (clubfoot)	History of bilateral clubfeet, which was operated on at the age of 6 years.
2. Characteristic craniofacial features, which are evident at birth or in early infancy	Absent
3. Characteristic cutaneous features, including skin hyperextensibility, easy bruising, skin fragility with atrophic scars, and increased palmar wrinkling	Presence of skin hyperextensibility and increased palmar creases
Minor criteria	
1. Recurrent/chronic dislocations	Absent
2. Pectus deformities (flat, excavated)	Absent
3. Spinal deformities (scoliosis, kyphoscoliosis)	Kyphoscoliosis present
4. Peculiar fingers (tapering, slender, cylindrical)	Arachnodactyly present
5. Progressive talipes deformities (valgus, planus, cavus)	Absent
6. Large subcutaneous hematomas	Hematoma over the left thigh
7. Chronic constipation	Absent
8. Colonic diverticula	Absent
9. Pneumothorax/ Pneumohemothorax	Absent
10. Nephrolithiasis/cystolithiasis	Absent
11. Hydronephrosis	Present detected incidentally on USG
12. Cryptorchidism in males	Present
13. Strabismus	Absent
14. Refractive errors (myopia, astigmatism)	Present
15. Glaucoma/ elevated intraocular pressure	Present for which underwent trabeculectomy at the age of 14 years.

Case one had autosomal recessive inheritance and fulfilled two major (congenital multiple contractures and characteristic cutaneous manifestations) and seven minor criteria (kyphoscoliosis, arachnodactyly, large subcutaneous hematoma, hydronephrosis, cryptorchidism, refractory errors, and glaucoma) and thus fulfilled the criteria for mcEDS. However, the patient did not have classic congenital craniofacial abnormalities (a major criterion) nor other minor criteria (recurrent/ chronic dislocations, pectus deformity, chronic constipation, colonic diverticula, pneumothorax, nephrolithiasis/cystolithiasis, and strabismus). A definite diagnosis could have been established by demonstrating mutations in the CHST14 gene encoding the D4ST1 enzyme or the DSE1 gene encoding dermatan sulfate epimerase (DSE). Both enzymes are involved in the biosynthesis of glycosaminoglycan (GAG) dermatan sulfate. However, most institutions do not have facilities to investigate genetic mutations, and outsourcing was beyond the financial capacity of the patient. As a caveat, even these tests can be false negatives.

Differential diagnoses included vascular EDS, hypermobile EDS, kyphoscoliotic EDS, and Marfan’s disease. Due to the history of recurrent hematoma and case reports of the association between cryptorchidism and vascular EDS, we initially considered a diagnosis of vascular EDS [5,6]. However, vascular EDS is autosomal dominant and is characterized by catastrophic vascular events and hollow visceral rupture, which did not match the clinical history of case one [4]. Kyphoscoliotic EDS (kEDS) seems highly probable due to its autosomal recessive inheritance, although we could not confirm by mutation studies, and the presence of early onset kyphoscoliosis, joint hypermobility, skin hyperextensibility, talipes equinovarus, and high myopia, which are consistent with kEDS [4]. However, the minimal criteria required for the classification of kEDS include the presence of congenital muscle hypotonia, which was absent in the index case. The case, although similar to Marfan syndrome, did not fulfil the Ghent criteria to be classified as Marfan syndrome [7]. Hence, referring to the most recent 2017 international classification of EDS, the clinical features of the subject best fit into the musculocontractural type of EDS by satisfying two major and seven minor criteria.

Patient one was sent home with advice to restrict outdoor activities because of the propensity for easy bruising and poor wound healing. Regular follow-up with an ophthalmologist and cardiologist was advised.

Case report two

A 34-year-old female presented with recurrent syncope. There was a history of frequent dislocation of the shoulders and hip joints. At the age of seven years, she had the first episode of spontaneous dislocation of the left shoulder, which was reducible. Owing to the increased frequency of spontaneous dislocations and associated pain, she underwent the Putti Platt procedure at the age of 29 years. She also had a history of an unstable right shoulder and right hip, which were often dislocated even at rest. Similar to case one, she also had kyphoscoliosis of the spine. There was no history of similar illnesses in the family.

**Figure 5:**
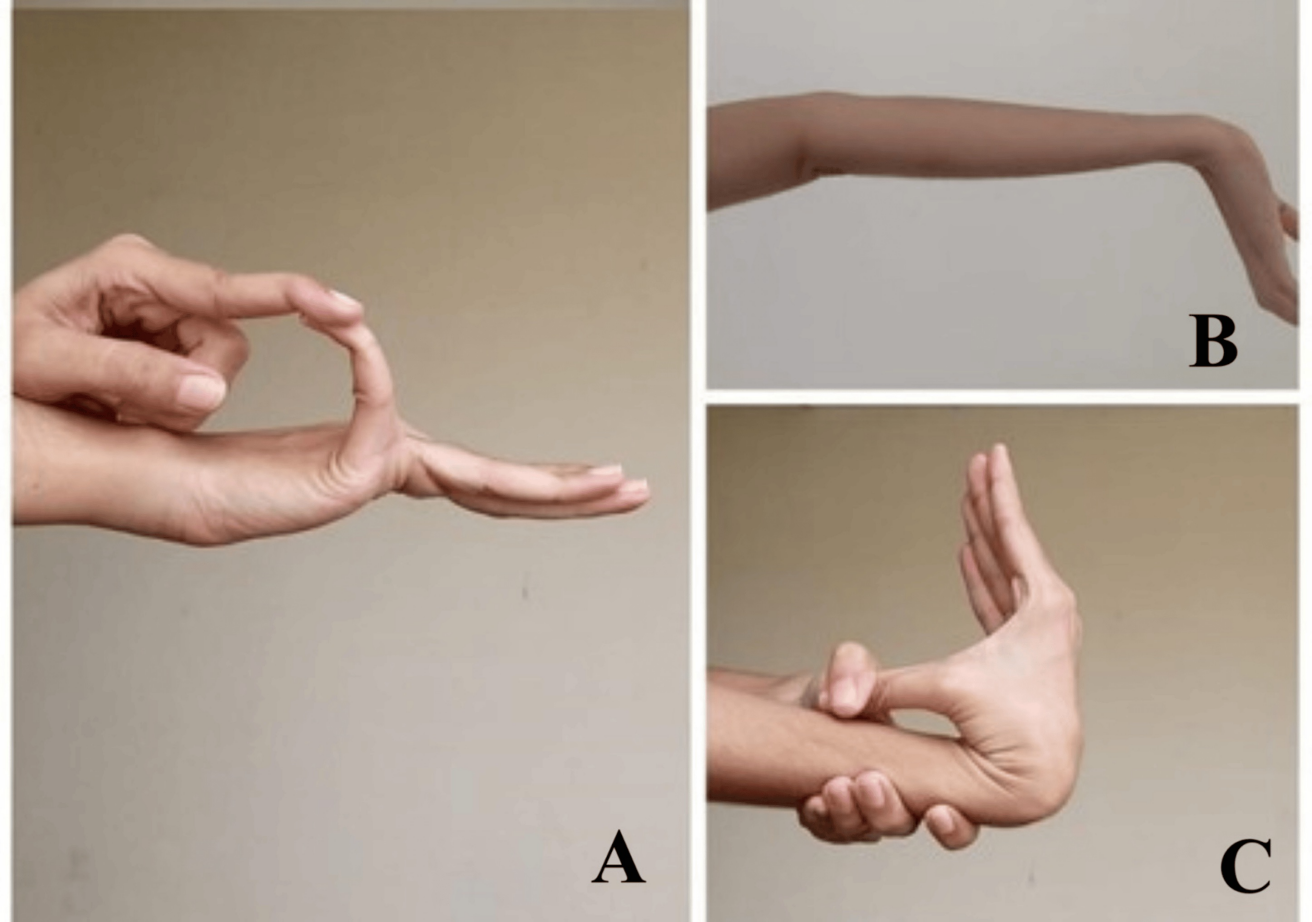
Case two generalized joint hypermobility A: passive dorsiflexion and hyperextension of the fifth MCP joint beyond 90°; B: passive hyperextension of the elbow beyond 10°; C: passive apposition of the thumb to the flexor aspect of the forearm.

Physical examination revealed soft, velvety skin and an arm-span to height ratio of 1.06. Musculoskeletal examination revealed generalized hypermobility (Figure 5), as suggested by a Beighton score of 6. None of the joints was tender or swollen. Routine blood tests revealed no major abnormalities (Table 3).

**Table 3:** Routine blood investigations of case two Results do not reveal any major abnormality. ESR: erythrocyte sedimentation rate; CRP: C-reactive protein; ALP: alkaline phosphatase; SGOT: serum glutamic-oxaloacetic transaminase; SGPT: serum glutamic-pyruvic transaminase; HDL: high-density lipoprotein; LDL: low-density lipoprotein.

Parameters	Value (normal range)
Hemoglobin	11.4g/dL (12-16g/dL)
White blood cell	8600/mm3 (4000-11000/mm3)
Differential count	Neutrophils 55% Lymphocytes 38% Eosinophils 6% Monocyte 1% Basophils 0%
Platelet count	3.4lacs/mm3 (1.5-4.5/mm3)
ESR (Westergren)	24 (0-20mm 1st hr)
CRP	0.306 (0-0.3mg/dL)
Blood urea	20 (7-20mg/dL)
Serum creatinine	0.6 (0.6-1.4mg/dL)
Serum sodium	139 (135-145meq/L)
Serum potassium	4.2 (3.5-5meq/L)
Fasting blood sugar	91 (70-100mg/dL)
Total bilirubin	0.9(0.1-1.2mg/dL)
Serum ALP	46 (44-147U/L)
SGOT	29 (10-40U/L)
SGPT	20 (10-40U/L)
Serum total protein	6.4 (6-8g/dL)
Serum albumin	3.5 (3.5-5g/dL)
Serum triglycerides	90 (50-150mg/dL)
Serum cholesterol	189 (125-200mg/dL)
Serum HDL	42 (30-65mg/dL)
Serum LDL	129 (85-150mg/dL)

**Figure 6:**
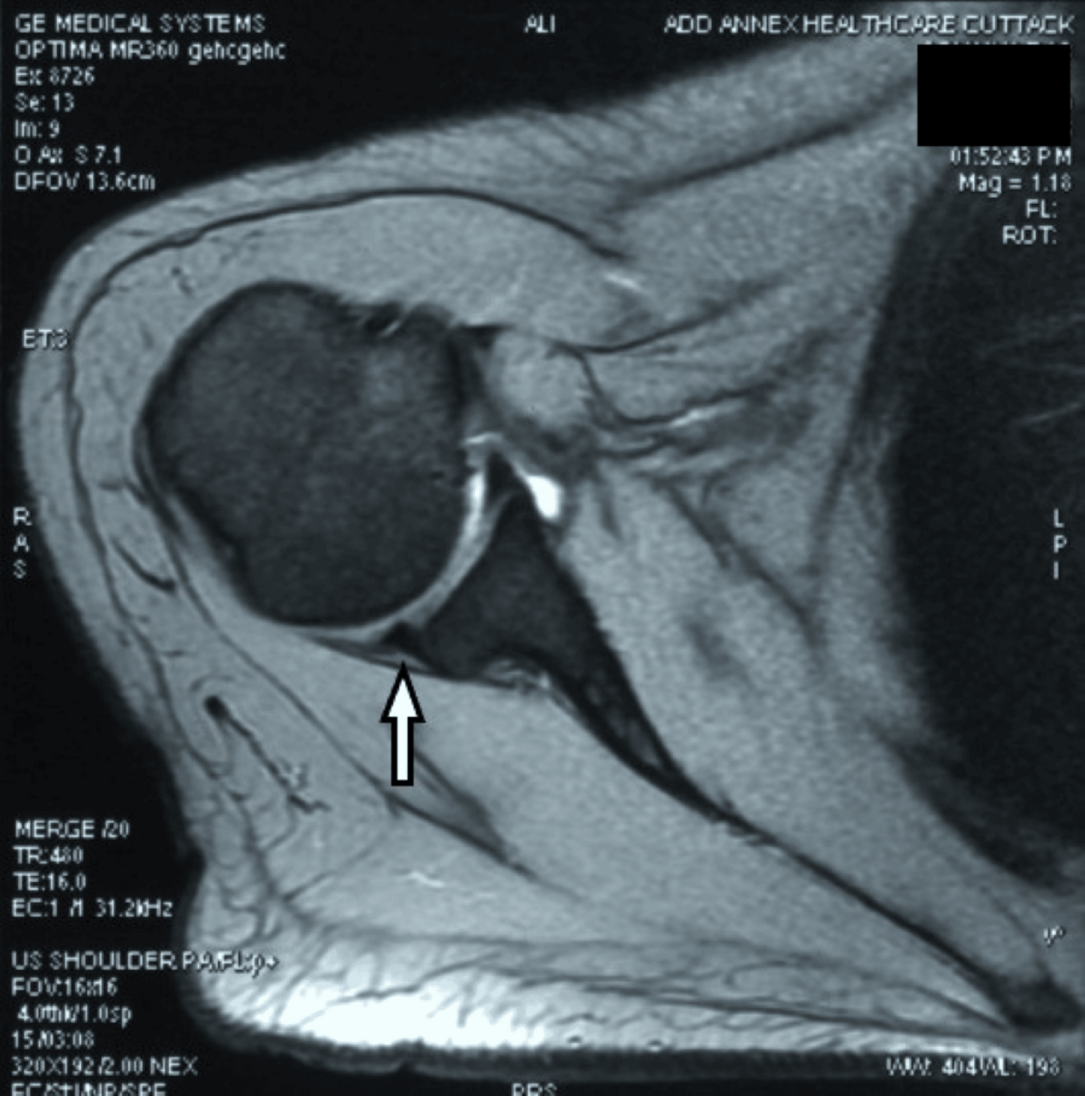
MRI scan of right shoulder for case two showing an anteroinferior labral tear, also known as “Bankart injury” (white arrow)

**Figure 7:**
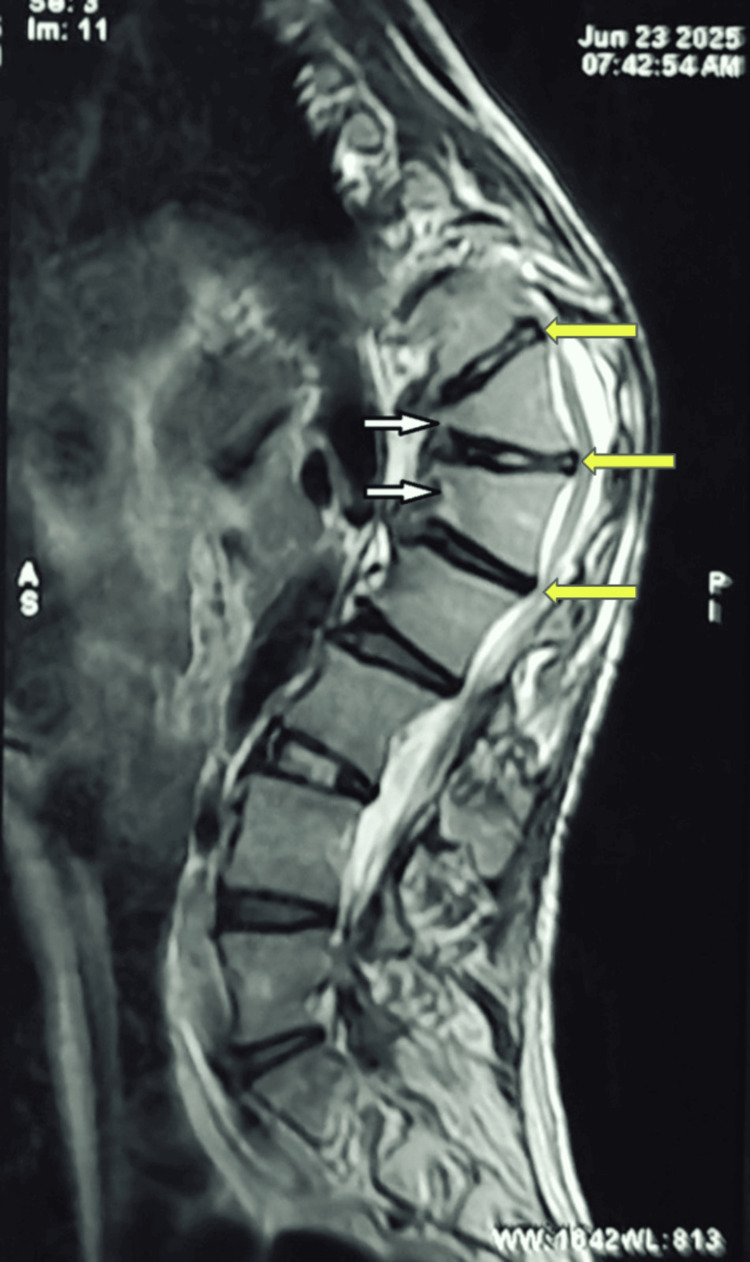
MRI spine for case two showing kypho-scoliotic deformity of dorso-lumbar spine D12 and L1 vertebral body shows anterior wedging (white arrows). D11-D12, D12-L1 and L1-L2 intervertebral discs show mild posterior disc contour bulge (yellow arrows) causing ventral thecal sac indentation

Her MRI scan revealed a Bankart injury in the right shoulder, with a shallow glenoid cavity (Figure 6), and MRI of both hips showed no abnormality. MRI of the spine revealed kypho-scoliotic deformity of the dorso-lumbar spine, with D12 and L1 vertebral body anterior wedging (Figure 7).

The patient was initially diagnosed as a case of hypermobility syndrome. For the past two years, the patient has also suffered from recurrent episodes of syncope, which have affected her daily life. Electrocardiograph, 2D Echocardiography, and 24-hour Holter monitoring revealed no significant abnormalities. The patient was subjected to a head-up tilt table test. The supine blood pressure at the onset of the test was 122/ 70 mm Hg, and the pulse rate of 86/minute. Then the patient was tilted at 75 degrees for 30 minutes. At the 30th minute, she had an episode of syncope followed by asystole, which reverted to normal rhythm post atropine and IV fluids. During this episode patient had symptoms of giddiness, blurring of vision, and blackout associated with a fall in blood pressure to 86/ 56 mm Hg. The pulse rate was 89/minute at the time of syncope and was similar to the initial pulse rate. The cardiologists concluded the test to be positive for combined cardioinhibitory and vasodepressor syncope.

Hypermobile EDS (hEDS) is another rare type of EDS characterized by generalized joint hypermobility without the characteristic skin manifestations seen in classical EDS [4]. It is associated with other systemic features, such as chronic pain, chronic fatigue, anxiety, depression, dysautonomia, dysphagia, constipation, osteoarthritis, and pelvic dysfunction. Overall, the quality of life of these patients is poor [8]. It is the only type of EDS without a known diagnostic molecular marker; hence, the diagnosis is purely based on clinical features [4]. Joint hypermobility syndrome and hypermobile EDS are clinically indistinguishable from each other; hence, they are presently considered part of the same disease spectrum. According to the 2017 International Classification System, all three of the following criteria must be fulfilled to diagnose hypermobile EDS [4]. These include 1) Generalized joint hypermobility; 2) Two or more of the following features: (a) systemic manifestations of generalized connective tissue disorder, (b) positive family history, and (c) musculoskeletal complications (chronic pain for >three months and/or recurrent joint dislocations/instability). 3) Absence of unusual skin fragility characteristic of other types of EDS and exclusion of other heritable connective tissue disorders and autoimmune rheumatologic conditions.

Patient two had generalized joint hypermobility, as evidenced by a Beighton score of 6. For the classification of hEDS, a Beighton score ≥ 5 out of 9 is required. Although she had some systemic manifestations of generalized connective tissue disorder, such as soft, velvety skin and increased arm-span-to-height ratio, at least 5 out of 12 must be fulfilled to satisfy the criterion. The patient had no family history. She had a definite history of recurrent joint dislocation that required surgical intervention. She did not have any features of skin fragility or any other rheumatological conditions. Therefore, her condition was diagnosed as hypermobile EDS.

## Discussion

Although Ehlers-Danlos syndrome is clinically and genetically heterogeneous, all subtypes share common features of joint hypermobility, skin hyperextensibility, and tissue fragility [1]. Eleven subtypes were described for the first time by the 1988 Berlin Nosology, and since then, with the discovery of new cases with variable genotypes and phenotypes, Ehlers-Danlos syndrome has been reclassified multiple times [2-4]. According to the 2017 International Classification System for Ehlers-Danlos Syndrome, there are 13 subtypes; each subtype has major and minor criteria and has defined genetic markers except for hypermobile EDS [4].

The prevalence of mcEDS is less than 1 in 1000,000 [9]. The rarity of the disease is exemplified by only four reported cases from India. In contrast, hEDS is a more common variant of Ehlers-Danlos syndrome, its prevalence ranging from one to five in 10,000 [10].

In mcEDS, large subcutaneous hematomas are a common and serious complication. Even trivial trauma can cause life-threatening hematomas that may lead to hemorrhagic shock, which requires intensive treatment. A study of 66 patients with mcEDS found that large subcutaneous hematomas were the most common vascular complication, seen in 46 of the 58 cases where this data was available. The first episode in most of these cases occurred by the age of 12 years [11].

Multiple case reports have described cardiac autonomic dysfunction in patients with hEDS, findings of which have been summarised in Table 4 [12-16].

**Table 4:** Presentation of Cardiac dysautonomia in hEDS. *Cardiovascular autonomic dysfunction was present in 21.6% patients including POTS in 16.4% and orthostatic intolerance in 5.2%. We presume the percentage calculation was done based on all patients included in the study. NMH: neurally mediated hypotension; OI: orthostatic intolerance; POTS: postural orthostatic tachycardia syndrome; OH: orthostatic hypotension.

Author	Type of study	No of patients with hEDS in the study	No of cases undergoing tilt table test	Type of cardiac dysautonomia on tilt- table test	No of patients with cardiac dysautonomia
Rowe et al. [12]	Cross- sectional	6	6	NMH and POTS	5
				NMH only	0
				POTS only	1
De Wandele et al. [13].	Case- control	80	39	OI	29
				POTS	16
Krahe et al. [14] *	Cross- sectional	117	Not mentioned	OI	6
				POTS	19
Gazit et al. [15]	Case- control	48	27	OI	11
				POTS	4
				OH	6
Celletti et al. [16]	Cross- sectional	102	99	OI	26
				POTS	49
				OH	4

The four main presentations of cardiac autonomic dysfunction described in hEDS are orthostatic intolerance (OI), orthostatic hypotension (OH), postural orthostatic tachycardia syndrome (POTS), and neurally mediated hypotension (NMH) [17]. The exact incidence and prevalence of the individual types are unknown. The most commonly described mechanism is the pooling of blood in dependent veins due to abnormal vascular laxity and venous distension, which reduces venous return to the heart and thus cardiac output, and is the primary mechanism behind OI, OH, and POTS [12]. However, this is associated with a sympathetic response that leads to reflex tachycardia. The tilt table test demonstrated the presence of mixed vaso-depressor and cardio-inhibitory syncope, which was associated with a reduced heart rate. This suggests that, in addition to vascular laxity, there was increased parasympathetic activity and reduced sympathetic activity. In other words, neurally mediated hypotension was active in the patient. In the absence of asystole, vasodepressors and cardio-inhibitory syncope are usually benign and require supportive therapy only. Various pharmacological options for the treatment of neurally mediated hypotension include midodrine, beta-blockers, and pyridostigmine.

The most important diagnostic challenge of Ehlers-Danlos syndrome is a lack of awareness about the clinical condition. The second diagnostic challenge is that most of the variants are progressive, and clinical suspicion before the evolution of the full clinical picture satisfying the classification criteria is challenging. Lastly, current classification criteria require mandatory genetic testing for almost all variants. In low- and middle-income countries, institutional genetic testing is sparsely available, and commercial affordability is a big challenge.

Limitations

Although the current International Classification System for Ehlers-Danlos Syndrome (2017) requires genetic testing for all EDS variants except the hypermobile variant (Case two), genetic testing was not available due to financial constraints. We are making efforts for Institutional access to genetic testing with government support. This will achieve definitive diagnosis and better patient care.

## Conclusions

The two cases highlight two subtypes of a rare disease: Ehlers-Danlos Syndrome-musculoskeletal EDS and hypermobile EDS. The first case highlights the possibility of a rare disease, such as musculo-contractural EDS, with a multi-systemic presentation. The second case reiterates the association between syncope and hypermobile EDS. This case also highlights cardiac autonomic dysfunction and neurally mediated hypotension as additional mechanisms for syncope. Larger queue verification is required.
